# High-Throughput Phenotyping of Sorghum Plant Height Using an Unmanned Aerial Vehicle and Its Application to Genomic Prediction Modeling

**DOI:** 10.3389/fpls.2017.00421

**Published:** 2017-03-28

**Authors:** Kakeru Watanabe, Wei Guo, Keigo Arai, Hideki Takanashi, Hiromi Kajiya-Kanegae, Masaaki Kobayashi, Kentaro Yano, Tsuyoshi Tokunaga, Toru Fujiwara, Nobuhiro Tsutsumi, Hiroyoshi Iwata

**Affiliations:** ^1^Laboratory of Biometry and Bioinformatics, Department of Agricultural and Environmental Biology, Graduate School of Agricultural and Life Sciences, The University of TokyoTokyo, Japan; ^2^Institute for Sustainable Agro-ecosystem Services, Graduate School of Agricultural and Life Sciences, The University of TokyoTokyo, Japan; ^3^Air4D Co., Ltd.Tokyo, Japan; ^4^Laboratory of Plant Molecular Genetics, Department of Agricultural and Environmental Biology, Graduate School of Agricultural and Life Sciences, The University of TokyoTokyo, Japan; ^5^Bioinformatics Laboratory, Department of Life Sciences, School of Agriculture, Meiji UniversityKanagawa, Japan; ^6^Earthnote Co., Ltd.Okinawa, Japan; ^7^Laboratory of Plant Nutrition and Fertilizers, Department of Applied Biological Chemistry, Graduate School of Agricultural and Life Sciences, The University of TokyoTokyo, Japan

**Keywords:** genomic prediction, high-throughput phenotyping, near-infrared (NIR), sorghum plant height, UAV remote sensing

## Abstract

Genomics-assisted breeding methods have been rapidly developed with novel technologies such as next-generation sequencing, genomic selection and genome-wide association study. However, phenotyping is still time consuming and is a serious bottleneck in genomics-assisted breeding. In this study, we established a high-throughput phenotyping system for sorghum plant height and its response to nitrogen availability; this system relies on the use of unmanned aerial vehicle (UAV) remote sensing with either an RGB or near-infrared, green and blue (NIR-GB) camera. We evaluated the potential of remote sensing to provide phenotype training data in a genomic prediction model. UAV remote sensing with the NIR-GB camera and the 50th percentile of digital surface model, which is an indicator of height, performed well. The correlation coefficient between plant height measured by UAV remote sensing (PH_UAV_) and plant height measured with a ruler (PH_R_) was 0.523. Because PH_UAV_ was overestimated (probably because of the presence of taller plants on adjacent plots), the correlation coefficient between PH_UAV_ and PH_R_ was increased to 0.678 by using one of the two replications (that with the lower PH_UAV_ value). Genomic prediction modeling performed well under the low-fertilization condition, probably because PH_UAV_ overestimation was smaller under this condition due to a lower plant height. The predicted values of PH_UAV_ and PH_R_ were highly correlated with each other (*r* = 0.842). This result suggests that the genomic prediction models generated with PH_UAV_ were almost identical and that the performance of UAV remote sensing was similar to that of traditional measurements in genomic prediction modeling. UAV remote sensing has a high potential to increase the throughput of phenotyping and decrease its cost. UAV remote sensing will be an important and indispensable tool for high-throughput genomics-assisted plant breeding.

## Introduction

Improving the throughput of phenotyping in the field is a big challenge in plant genetics, physiology, and breeding. The emergence of the next-generation sequencing technologies enables us to obtain genome-wide DNA polymorphism data for a large number of samples easily and rapidly (Mardis, 2007; Davey et al., 2011). Statistical methods, such as genome-wide association study (GWAS; Brachi et al., 2011; Korte and Farlow, 2013; Huang and Han, 2014) and genomic selection (GS; Meuwissen et al., 2001; Jannink et al., 2010) allow us to associate DNA polymorphism data, which is extremely high-dimensional, to phenotypic variations in agronomic traits. Boosted by these technological developments, the efficiency of plant breeding is expected to improve rapidly (Huang and Han, 2014). However, phenotyping is still time consuming and labor intensive, and may be more costly than genotyping. Thus, phenotyping has become a serious bottleneck in the acceleration of plant breeding (Furbank and Tester, 2011). Field experiments at multiple plant breeding stations over a large geographic area are indispensable to evaluate the adaptability of new candidate genotypes and to examine the pattern of genotype-environment interaction (Chapman et al., 2014). At each breeding station, a large number of genotypes are tested in the field. Most of the measurements conducted in the field are destructive and labor- and time-intensive, and thus cannot be repeated frequently in the course of plant growth. Because phenotypic data is necessary for genomics-assisted breeding, it is the first priority to develop a high-throughput phenotyping method.

Remote sensing using a low-altitude unmanned aerial vehicle (UAV), such as radio-controlled multicopter, can solve the problem described above. Besides low-altitude UAVs, measurements using satellites (Inoue, 1997) and ground-based vehicles (Lee and Searcy, 1999) have been applied for remote sensing of growth conditions of crop plants (Sugiura et al., 2005). However, satellites have low resolution, poor sensitivity under cloudy conditions, and slow data transmission (Sugiura et al., 2005; Zhang and Kovacs, 2012), and ground-based vehicles cannot enter fields with tall crops or muddy soil (Sugiura et al., 2005; Chapman et al., 2014). Low-altitude UAVs have no such disadvantages and can be used without expert skills (Merz and Chapman, 2011). Most low-altitude UAVs have an autopilot function to fly automatically along a route designed by mission planning software (Berni et al., 2009; Chapman et al., 2014; Zarco-Tejada et al., 2014; Díaz-Varela et al., 2015). Another widespread remote sensing technology is light detection and ranging (LiDAR). However, this technology has some shortcomings, e.g., high cost of data acquisition and processing (Díaz-Varela et al., 2015). The emergence of computer-vision technologies, such as the structure-from-motion and multi-view-stereo algorithms, enables reconstruction of accurate 3D-models from a series of images with a considerable overlap between adjacent images. These technologies are attractive alternatives to LiDAR, due to their high performance, flexibility, and relatively low cost (Díaz-Varela et al., 2015). For remote sensing of plants, near-infrared (NIR) cameras have been used in many studies (Lee and Searcy, 1999; Sugiura et al., 2005; Berger et al., 2010; Cabrera-Bosquet et al., 2011; van Maarschalkerweerd et al., 2013; Colomina and Molina, 2014; Díaz-Varela et al., 2015; Torres-Sánchez et al., 2015), because plant leaves (or chlorophylls) strongly reflect NIR light (Knipling, 1970; Tucker, 1979; Fahlgren et al., 2015) and some indices based on NIR reflectance rate, such as normalized difference vegetation index (NDVI; Rouse et al., 1974), are useful for identifying plants and assessing their growing conditions via remote sensing. Some studies have indicated that NIR sensors have advantages over standard RGB sensors in plant monitoring (Nijland et al., 2014; Zhang et al., 2016). Nevertheless, NIR cameras are less common, and often more expensive than RGB cameras (or extra cost is needed to modify RGB cameras into NIR cameras). Because remote sensing is a promising tool for phenotyping, we compared the advantages of RGB and NIR cameras in phenotyping and genomic prediction modeling.

Genomic selection is a novel breeding method that allows selection of complex traits with genome-wide markers. Because the selection is performed on the basis of the genetic potential predicted from these markers, GS requires building an accurate prediction model based on a dataset of individuals or lines that have been genotyped and phenotyped (Meuwissen et al., 2001; Jannink et al., 2010). A large dataset is needed to build an accurate prediction model. As mentioned above, however, phenotyping is time consuming and labor intensive, and is a serious bottleneck in building an accurate model. If UAV remote sensing can streamline the collection of phenotypic data, it will greatly enhance the potential of GS.

Using image-processing software for photogrammetry, we can obtain ortho-mosaic and a digital surface model (DSM) from UAV images (Gini et al., 2013). Ortho-mosaic is a distortion-corrected image. DSM provides information on the altitude. In plant science, DSM information has been applied to estimate biomass and plant height of barley (Bendig et al., 2014), and plant height, volume, and canopy size of olive trees (Zarco-Tejada et al., 2014; Díaz-Varela et al., 2015; Torres-Sánchez et al., 2015). Currently, we are using genomics-assisted breeding to develop a sorghum [*Sorghum bicolor* (L.) Moench] variety that can be used for high bioethanol production for biofuel. Plant height is one of the most important traits affecting bioethanol yield. Because some sorghum accessions may be taller than 4 m, they are usually cut for measurements, which are labor intensive, whereas GS requires phenotypic data and marker genotype data for a large number of accessions.

The objectives of this study are the validation of the usefulness of UAV remote sensing for measurement of sorghum plant height and for genomic prediction modeling. First, we confirmed the accuracy of plant height estimates from UAV images under the conditions of small plot size. Next, we examined the accuracy of genomic prediction of plant height trained by UAV remote sensing data and data manually measured with a ruler. To evaluate the robustness of this method to plant height variation related to environmental differences such as nutrition level, sorghum plants were grown at two levels of nitrogen availability. We also compared the measurement and prediction accuracy of different cameras and different procedures of remote sensing data analysis.

## Materials and Methods

### Field Experiment

**FIGURE 1:**
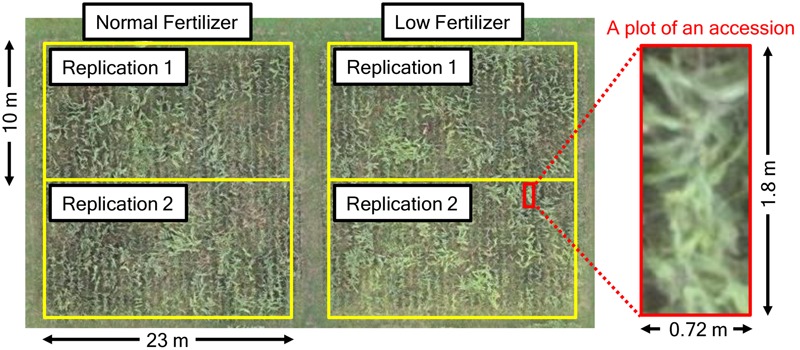
**Field design in this study**.

In this study, we used 172 accessions from sorghum germplasm collections (Supplementary Table 1). Of these, 78 accessions were from the world core collection of NIAS (National Institute of Agrobiological Science, Ibaraki, Japan; integrated into the National Agriculture and Food Research Organization from April 1, 2016), 91 were from the sorghum mini core collection of ICRISAT (International Crops Research Institute for the Semi-Arid Tropics, Patancheru, India) and 3 were original cultivars developed by EARTHNOTE Co., Ltd. (Okinawa, Japan). Seeds were sown on 200-cell plug trays on May 8, 2014. Seedlings were transplanted to a field of the Institute for Sustainable Agro-ecosystem Services, the University of Tokyo (Tokyo, Japan; 35°44′09.1″N, 139°32′23.7″E, 60 m above the sea level) on June 9, 2014. An outline of the field design is shown in **Figure 1**. To investigate the effect of fertilization on plant growth, we used normal (N-P-K: 1.2-1.8-1.6 kg/a) and low (N-P-K: 0.6-1.8-1.6 kg/a) fertilization in two replications per treatment per accession (172 × 4 = 688 plots in total). Five plants were grown in each plot (inter-plant spacing, 0.3 m; inter-row spacing, 0.72 m). On October 2 and 3, two plants per plot were harvested and their height was measured with a ruler. In total, 688 × 2 = 1,376 plants were measured.

### Remote Sensing Experiment

**FIGURE 2:**
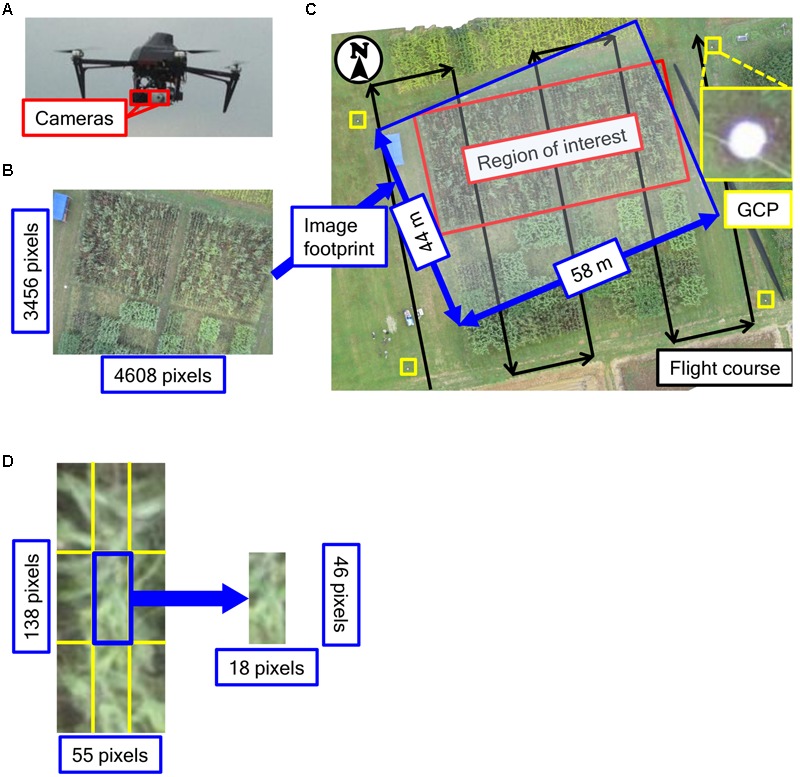
**Unmanned aerial vehicle used. (A)** Images of UAV used in the study, **(B)** An image taken in this study, **(C)** Outline of the flight course and **(D)** The size of one plot and one divided block. Positions of GCPs in the World Geodetic System were measured by using GPS.

The radio-controlled quadcopter USM-S1 (Air4D Co., Ltd., Tokyo, Japan; **Figure 2A**), was used as a UAV for remote sensing. Two digital cameras, Canon PowerShot ELPH 110HS (Canon Inc., Tokyo, Japan), were installed on the UAV (resolution, ca. 16.1 million pixels; sensor size, 6.2 mm × 4.7 mm; focal length, 4.3–21.5 mm). One was a normal RGB camera, and the other one was modified to capture NIR, green and blue (NIR-GB). The NIR-GB camera was purchased at MaxMax Inc. (Carlstadt, NJ, USA). The focal length was set at 4.3 mm. The focus was adjusted by the camera auto-focus function. On the ground at each corner of the field, we installed a white acrylic disk (27 cm in diameter) as a ground control point (GCP) (**Figure 2C**). The positions of GCPs in the World Geodetic System were measured by using GPS (Geo7X, Trimble Inc., Sunnyvale, CA, USA) and used in image processing.

To compare plant height measured with the UAV (PH_UAV_) and plant height measured with rulers (PH_R_), we performed a remote-sensing experiment on the first day of harvest (October 2). The weather on that day was cloudy. ISO sensitivity, which is an indicator of light sensitivity provided by the International Organization for Standardization (Vernier, Geneva, Switzerland), was set at 320 and shutter speed at 1/1,250 s for the RGB camera; ISO sensitivity was 800 and shutter speed was 1/800 s for the NIR-GB camera. The UAV was controlled by an autopilot system with GPS to fly along a pre-defined course designed by the PC Ground Station software (DJI Co., Ltd., Shenzheng, China). The outline of the flight course is shown in **Figure 2B**. The course was designed so that the vertical overlap of images was 70% and horizontal overlap was 30%. Photographs were taken at an altitude of 40 m, total flight time was about 10 min, and 30 photographs were taken. From an altitude of 40 m, the resolution was ca. 13 mm per pixel. A preliminary remote sensing experiment was performed on July 23 with the RGB camera NEX-7 (Sony Corporation, Tokyo, Japan; resolution, ca. 24 million pixels; sensor size, 23.5 mm × 15.6 mm) with a lens of focal length 20 mm. The following parameters were used in the preliminary experiment: ISO sensitivity, 100; shutter speed, 1/800 s; altitude, 50 m; 78 photographs were taken. From an altitude of 50 m, the resolution was ca. 10 mm per pixel. Because plants were still small on June 23, we used the data collected on July 23 to obtain DSM data on the ground surface of the field. Although NEX-7 has higher resolution than PowerShot ELPH 110HS, it is heavier and thus we could not mount two types of cameras (RGB and NIR) simultaneously on the UAV. At an altitude of 40 m, the resolution of PowerShot ELPH 110HS was similar to that of NEX-7. Therefore, we used PowerShot ELPH 110HS for remote sensing.

### Image Processing

Both RGB and NIR-GB images were analyzed in the same way. By using the in-house structure-from-motion software Nadir-metry (Air4D Co., Ltd., Tokyo, Japan), ortho-mosaic images and DSM data were constructed from images taken by the UAV with the geographic coordinates of GCPs. Although the structure-from-motion algorithm of Nadir-metry is similar to that in other software, it has some advantages in feature point matching and generating point clouds. In the algorithm of Nadir-metry, feature point matching is performed by taking into account the correspondence between overlapping images estimated from their geographic coordinates. As a result, spatial skew hardly occurs. In the generation of point clouds, all pixels were analyzed to detect matching points. This decreased the number of missing matches and prevented point clouds from being sparse. Because DSM values were calculated based on the World Geodetic System 1984 and they did not directly reflect the ground height of objects, we estimated the height of sorghum plants by subtracting the DSM values of the ground surface of the field on July 23 from the DSM values on October 2, as in Bendig et al. (2013). From the location of each plot determined on the ortho-mosaic image, we obtained PH_UAV_ for each plot from the DSM data. Because adjacent plots were close to each other, DSMs of plot boundaries were contaminated with data originated from adjacent plots, and might have higher error than those inside a plot. To exclude marginal areas, we divided each plot into 9 blocks (3 × 3) and analyzed only the central block. That is, the plot size was 0.72 m × 1.8 m and corresponded to ca. 55 × 138 pixels (**Figure 2D**) at a resolution of 13 mm per pixel in DSM. The size of the central block was 0.24 m × 0.6 m, corresponding to ca. 18 × 46 pixels of DSM (**Figure 2D**). Each pixel had a DSM value that was construed as the altitude of the location. We calculated the 50th (median), 75th, 90th, and 99th percentiles of DSM values of the central block as the representative values of PH_UAV_ for the plot. We evaluated the accuracy of PH_UAV_ from its correlation coefficients with PH_R_ and also from root mean square difference (RMSD):

$$\text{RMSD}=\sqrt{\frac{1}{688}\sum_{i=1}^{688}{\left({\text{PH}}_{\text{UAV},i}-{\text{PH}}_{\text{R},i}\right)}^{2},}$$

where PH_UAV_, *_i_* and PH_R_, *_i_* are the PH_UAV_ value and the PH_R_ value of the *i*th plot, respectively.

### Treatment of Unexpected Values

We assumed that if a plot of a tall accession and that of a small accession were adjacent in the field, the plants of the small accession might be overlapped by those of the tall accession on images, and DSMs of the small accession might be overestimated because they were strongly contaminated by data originated from the tall accession. To confirm this, we compared the correlation between PH_UAV_ and PH_R_ under three conditions: (1) using data of the replication with higher PH_UAV_ value for each accession; (2) using data of the replication with lower PH_UAV_ value for each accession; (3) using average PH_UAV_ value of two replications for each accession. As described later, the result using the lower PH_UAV_ was better than others, and the replication with lower PH_UAV_ value for each accession was used in the following analysis.

### Genomic Prediction Modeling

We built genomic prediction models for both PH_UAV_ and PH_R_, and compared the predicted values. To obtain DNA polymorphism data, we used restriction site-associated DNA sequencing (RAD-Seq; Baird et al., 2008); which is cheaper than whole genome sequencing especially for analysis of DNA polymorphism of large number of accessions. We obtained the data for 66,132 SNPs in 151 accessions (Supplementary Table 1). Genomic best linear unbiased prediction (G-BLUP) using rrBLUP (Endelman, 2011) was used for the modeling. To calculate predicted values for the 151 accessions and compare them for PH_UAV_ and PH_R_, we performed leave-one-out cross-validation. In cross-validation, a model built with the PH_UAV_ or PH_R_ data of 150 of the 151 accessions was used to predict the PH_UAV_ or PH_R_ values of the remaining accession from its DNA polymorphism data. By comparing the observed and predicted values of both PH_UAV_ and PH_R_, we evaluated whether manual measurements (PH_R_) can be replaced with the measurements using UAV remote sensing (PH_UAV_) in the collection of data for building a model.

## Results

### Image Processing and Measurement of Plant Height via UAV Remote Sensing

**FIGURE 3:**
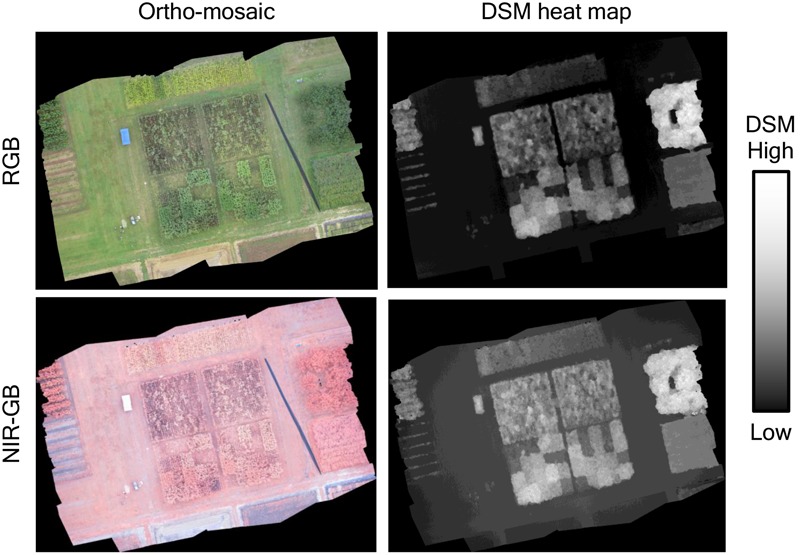
**Ortho-mosaic and DSM heat map of October 2.** Upper images were originated from the standard RGB cameras and lower images were originated from the NIR-GB (near-infrared, green, blue) camera. DSM provides information of the altitude. The resolution was ca. 13 mm per pixel. The sizes of both the ortho-mosaic and DSM were 10,701 × 8,061 pixels in RGB and 10,749 × 8,041 in NIR-GB; the ortho-mosaic and DSM were built from 30 images (4,608 × 3,456 pixels each).

For both RGB and NIR-GB cameras, ortho-mosaic images and DSM heat maps of the experimental field before harvest were constructed by using 30 remote-sensing images taken on October 2 (**Figure 3**).

**FIGURE 4:**
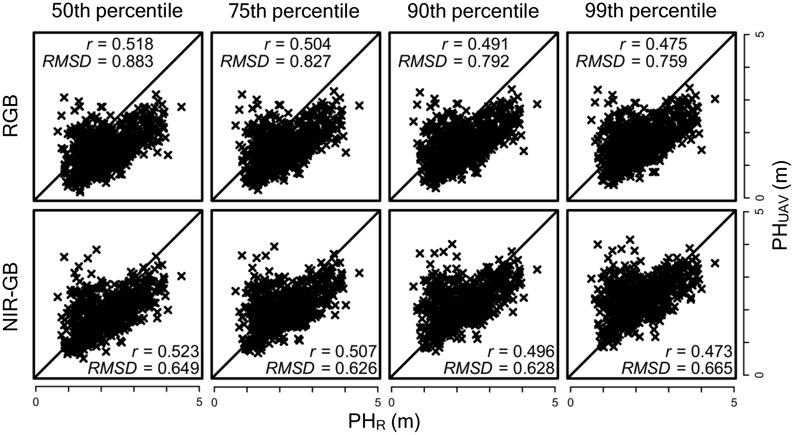
**Accuracy under different cameras and different percentiles of DSM.** The diagonal line indicates *y* = *x*; *r*, correlation coefficient; RMSD, root mean square difference. PH_UAV_ is the plant height measured with the UAV and PH_R_ is that with rulers. PH_UAV_ values were calculated as 50th, 75th, 90th, and 99th percentile of DSM values in each plot.

To assess the accuracy of plant height measurements, we compared the correlation between PH_UAV_ and PH_R_ obtained with the two cameras at the 50th, 75th, 90th, and 99th percentiles of DSM values (**Figure 4**). Although there was no significance between two correlation coefficients (RGB vs. NIR-GB) at any combinations, correlation coefficients were higher for the NIR-GB than for RGB camera: 0.523 vs. 0.518 (50th percentile), 0.507 vs. 0.504 (75th), and 0.496 vs. 0.491 (90th). However, correlation coefficients were higher for RGB (0.475) than for NIR-GB (0.473) at the 99th percentile. RMSD between PH_UAV_ and PH_R_ was lower for the NIR-GB than RGB camera: (0.649 vs. 0.883 (50th percentile), 0.626 vs. 0.827 (75th), 0.628 vs. 0.792 (90th), and 0.665 vs. 0.759 (99th). PH_UAV_ obtained from the RGB camera underestimated PH_R_ because the points were distributed below the *y* = *x* line in **Figure 4**. PH_UAV_ obtained from the NIR-GB camera estimated PH_R_ more accurately, especially at the 90th and 99th percentiles, as evidenced by smaller RMSD in RGB than in NIR-GB.

The relationship between PH_UAV_ and PH_R_ suggested that PH_UAV_ was overestimated at PH_R_ values of <2.0 m (**Figure 4**). To assess whether the presence of taller plants on adjacent plots resulted in overestimation, we calculated two types of ratio for each plot as follows:

$$r_{1}=\frac{\text{max}\{{\text{PH}}_{\text{R},i,k}|k=1,2,...,N\}}{{\text{PH}}_{\text{R},i}},r_{2}=\frac{{\text{PH}}_{\text{UAV},i}}{{\text{PH}}_{\text{R},i}}$$

where PH_R_, *_i_* and PH_UAV_, *_i_* are the PH_R_ and PH_UAV_ values of the *i*th plot, respectively, and *N* = 8 in this case. PH_R_, *_i, k_* is the PH_R_ value of the *k*th (1–8) plot adjacent to the *i*th plot. The ratio *r*_1_ represents the degree of height difference among the plants on adjacent plots. The ratio *r*_2_ represents the degree of over- or under- estimation of PH_UAV_ against PH_R_. A scatter plot of these ratios for one combination (NIR-GB camera and the 50th percentile of DSM values) is shown in Supplementary Figure 1A; the results for other combinations were similar. Theoretically, the PH_UAV_/PH_R_ ratio is expected to be approximately constant if UAV measurements are accurate, because both PH_UAV_ and PH_R_ are expected to be the true values of plant height (with measurement errors). If we regarded PH_R_ as the true plant height, the PH_UAV_/PH_R_ ratio became large, i.e., the PH_UAV_ was overestimated against PH_R_ (Supplementary Figure 1A) when PH_R_ on adjacent plots was 1.5 times that on the central plot. This result suggests that the presence of taller plants on an adjacent plot prevents accurate construction of the DSM of the target plot because of the overlapping effect.

**FIGURE 5:**
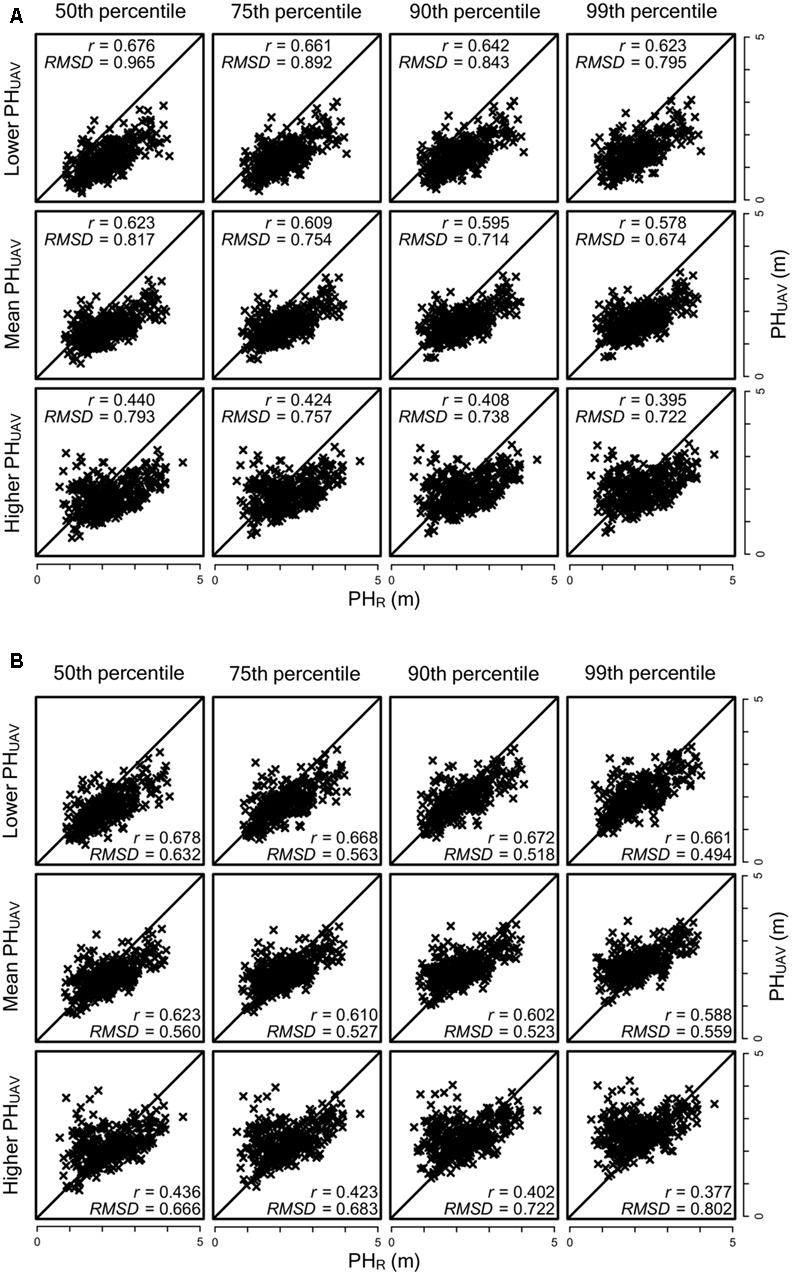
**Comparison of lower, high and average PH_UAV_ from two replications. (A)** RGB camera and **(B)** NIR-GB camera. The diagonal line indicates *y* = *x*; *r*, correlation coefficient; RMSD, root mean square difference.

We analyzed the relationships between PH_UAV_ and PH_R_ for all the combinations of the two cameras and four percentile values separately for each of two replications (one with the lower PH_UAV_ value, the other one with the higher PH_UAV_ value) and the average (**Figure 5**). For all combinations, the correlation coefficients were highest (around 0.65) for the replications with lower PH_UAV_ values and lowest (around 0.40) for the replications with higher PH_UAV_ values. Correlation coefficients with lower PH_UAV_ were significantly higher than those with mean PH_UAV_ at significant level of 10% at all combinations. This result suggested that the lower PH_UAV_ values were more reliable.

### Genomic Prediction Modeling

**FIGURE 6:**
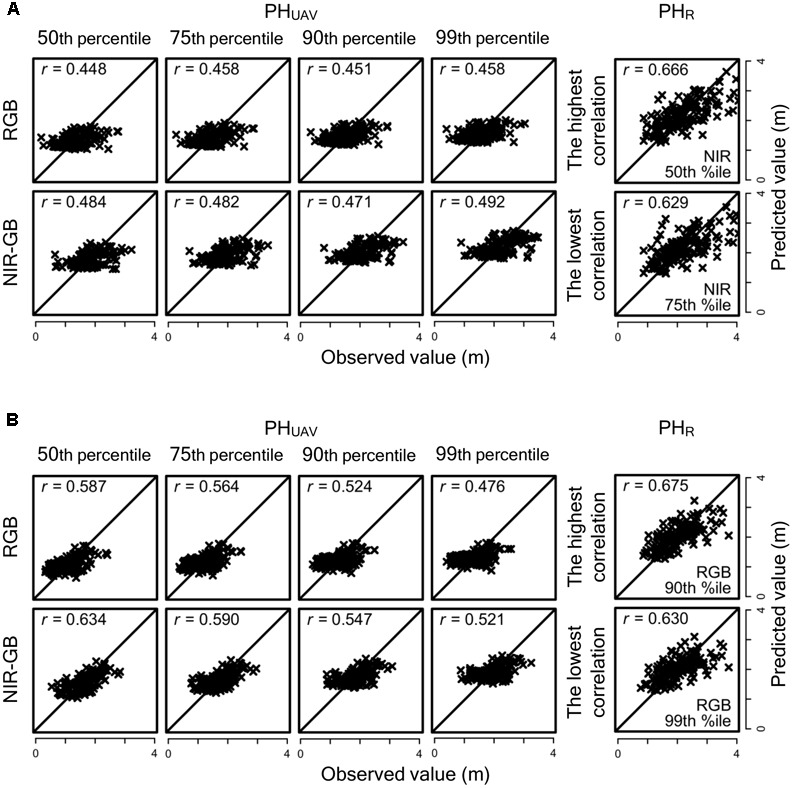
**Relationships between observed and predicted PH_UAV_ and PH_R_ values in genomic prediction under normal and low fertilization conditions. (A)** Normal fertilization and **(B)** Low fertilization. *r*, correlation coefficient. For PH_R_, only the combinations the highest and lowest correlation coefficients are shown.

**FIGURE 7:**
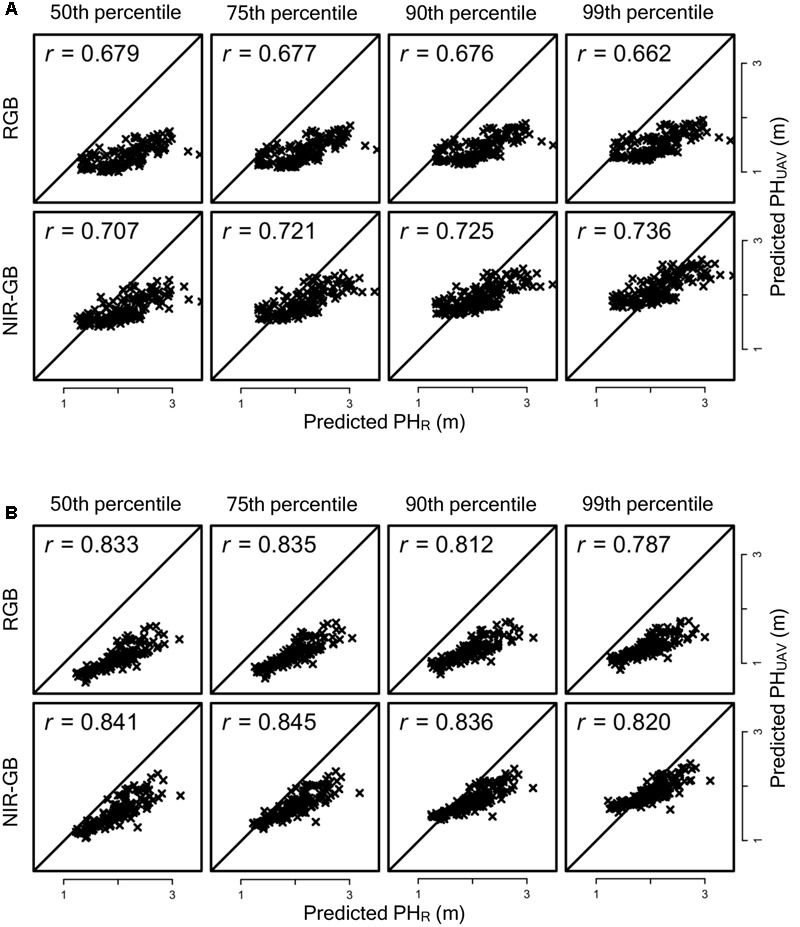
**Relationships between predicted PH_R_ and predicted PH_UAV_ under normal fertilization and low fertilization condition. (A)** Normal fertilization and **(B)** Low fertilization. *r*, correlation coefficient.

To evaluate the accuracy of genomic prediction for PH_UAV_ and PH_R_ and the agreement between their predicted values, we obtained these values via leave-one-out cross- validation for all sorghum accessions cultivated under normal and low fertilization conditions. The relationships between the observed and predicted PH_UAV_ and PH_R_ values are shown in **Figure 6**. If the combination of two cameras and four representative values is different, the combination of selected replications for each fertilization condition is also different. For example, we consider two combinations: (i) (PH_UAV_ of replication 1) < (PH_UAV_ of replication 2) for accession A and (PH_UAV_ of replication 1) < (PH_UAV_ of replication 2) for accession B in a combination of two cameras and four percentiles of DSM values (combination 1), and (ii) (PH_UAV_ of replication 1) < (PH_UAV_ of replication 2) for accession A and (PH_UAV_ of replication 1) > (PH_UAV_ of replication 2) for accession B in another combination (combination 2). Then, for accession A, replication 1 is selected as the plot with the lower PH_UAV_ value in both combinations, whereas for accession B replication 1 is selected in combination 1 and replication 2 is selected in combination 2 as the plot with lower PH_UAV_ value. The same combinations of replications were also used for PH_R_. However, the combinations of replications were almost the same between different combinations of two cameras and four percentiles. Because of this, the results of PH_R_ prediction with different cameras and different percentiles were similar to each other. The combinations with the highest and the lowest correlation coefficients are shown in **Figure 6** for PH_R_ prediction. Under normal fertilization, correlation coefficients between observed and predicted PH_UAV_ were less than 0.5 (range, 0.448–0.492) for all combinations of the type of cameras and the percentiles of DSM, whereas those for PH_R_ ranged from 0.629 to 0.675. Correlation coefficients for PH_UAV_ were higher under low fertilization than under normal fertilization in all combinations. Although almost all combinations had lower correlation coefficients for PH_UAV_ than for PH_R_, the correlation coefficient for PH_UAV_ in the combination of NIR-GB and 50th percentile was as high as that for PH_R_ (**Figure 6**). Predicted PH_UAV_ and predicted PH_R_ highly correlated with each other in all combination, (correlation coefficients ≥ 0.66 under low fertilization and ≥0.78 under normal fertilization except for one combination; **Figure 7**).

## Discussion

We introduced UAV remote sensing for high-throughput measurement of sorghum plant height, and applied it to genomic prediction modeling. The results of this study suggest the potential of UAV remote sensing for the high-throughput phenotyping of plant height in sorghum. Some sorghum genotypes are too tall to measure the height of the plants without harvesting them. Our approach would not only decrease labor cost but would also allow observation of plant growth over time. Traditionally, all accessions are measured only once, although their growth stages at the measurement time may differ. Our remote sensing approach would allow comparison of accessions at the same growth stage.

Using UAV remote sensing, we could not measure sorghum plant height as accurately as that of barley (which is smaller than sorghum) measured by Bendig et al. (2014). One reason may be that tall sorghum plants overlap (Supplementary Figure 1). Another reason may be low plant density: 3.9 plants/m^2^ vs. 300 plants/m^2^ in Bendig et al. (2014); we can see the sparseness of plants in a plot in **Figure 1**. If plant density is high enough to form a continuous canopy, most of the matched points are captured from the canopy and DSM reflects plant height precisely. However, because of the sparseness, matched points included not only the top of canopy but also the ground or lower parts of plants. This could cause errors on DSM in plant height measurement. Increasing plot size and plant density may improve the measurement accuracy. We can easily measure plant height multiple times with UAV remote sensing, which would probably also reduce measurement error. An important point is that the predicted PH_UAV_ values were highly correlated with the predicted values of PH_R_, even though the correlation between the observed values of PH_UAV_ and PH_R_ was not high. This is because the observed values of PH_UAV_ and PH_R_ had different types of errors (e.g., errors from manual measurements and UAV measurements). Higher correlation between the predicted values suggests that they were less affected by errors than observed values. If we can reduce the measurement errors of UAV remote sensing by improving technologies or by repeating measurements, UAV remote sensing will perform better than manual measurements for genomics-assisted breeding.

We also found that using the lower value of PH_UAV_ of the two replications for each association seemed to reduce the overlapping effect (**Figure 5**). For the replications with the lower value PH_UAV_ values, the correlation coefficients of PH_UAV_ and PH_R_ were not largely different for the RGB and NIR-GB cameras. The RGB camera underestimated PH_UAV_ and the RMSD value of this camera was higher than that of the NIR-GB camera. For genomic prediction modeling, the correlation coefficients between observed and predicted values were higher for NIR-GB than RGB (**Figures 6, 7**), indicating that NIR-GB was slightly superior for this purpose. Only the combination of NIR-GB and 50th percentile under low fertilization resulted in a correlation coefficient between observed and predicted values for PH_UAV_ close to that for PH_R_ (**Figure 6**). Prediction of plant height was less accurate under normal than under low fertilization. This may suggest that the overlapping effect was stronger when plants were larger. NIR-GB was better than RGB under both fertilization conditions. Like in crop identification (Zhang et al., 2016) and in monitoring of plant condition (Nijland et al., 2014), a NIR sensor may perform better than a standard RGB sensor for remote sensing in plant breeding.

In the case of the NIR-GB camera, the correlation coefficient was the highest at the 50th percentile of DSM values, whereas the RMSD value was the lowest at the 99th percentile. At the 99th percentile, PH_UAV_ was almost the same as PH_R_. RMSD became higher, but the number of outliers also increased, which reduced the correlation coefficient. At the 50th percentile, the number of outliers was low in PH_UAV_ measurements and the correlation coefficient became higher, but the difference between the PH_UAV_ and PH_R_ values increased and RMSD became lower. There is probably a trade-off between the correlation coefficient and RMSD. If accurate plant height measurements are required, PH_UAV_ at the 99th percentile of DSM will perform well. In this study, PH_UAV_ at the 50th percentile of DSM was better than at other percentile values regarding the accuracy of both plant height measurements and genomic prediction modelings.

The collection of PH_UAV_ data required 3 people × 10 min, while collection of PH_R_ data required over 10 people × 2 days. Because GS and GWAS require phenotyping of a large number of accessions or plants, UAV remote sensing will be an important and indispensable tool for high-throughput genomic-assisted plant breeding.

Using digital cameras, we can measure canopy cover from images taken right above the plants (Purcell, 2000) and relate it to plant density, early vigor, leaf size, and radiation interception (Liebisch et al., 2015). Using NIR sensors, we can measure NDVI of the canopy and relate it to canopy biomass and nitrogen status (Hansen and Schjoerring, 2003). Using thermal sensors, we can measure canopy temperature (Berni et al., 2009) and relate it to water stress (Jackson et al., 1981).

Not all of the studies used UAV remote sensing. However, by attaching appropriate sensors to an UAV, we can obtain various types of information from plants grown in the field and measure important target trait-related characteristics. Combination of machine learning and image analysis enables the evaluation of complex traits, such as flowering date (Guo et al., 2015). In the future, various kinds of plant phenotyping data will be measured in parallel and in a high-throughput manner by UAV remote sensing.

## Author Contributions

KW, TF, NT, and HI planned and designed the experiments. TT and NT contributed to the preparation of materials. KW, WG, and KA performed remote sensing experiments. HT performed the RAD-Seq experiment, and MK, HK-K, and KY analyzed the RAD-Seq data. KW performed other analyses, interpreted results, and drafted the manuscript with the contributions from WG, KA, and HI. NT obtained funding for the study. HI supervised the study. All authors read and approved the final manuscript.

## Conflict of Interest Statement

The authors declare that the research was conducted in the absence of any commercial or financial relationships that could be construed as a potential conflict of interest.
