# Supplementary material for: High-Throughput Phenotyping of Sorghum Plant Height Using an Unmanned Aerial Vehicle and Its Application to Genomic Prediction Modeling
Source: Front Plant Sci. 2017 Mar 28;8:421. doi: 10.3389/fpls.2017.00421 (PMC5368247; doi:10.3389/fpls.2017.00421)
Supplement: Supplementary file 1 [file Data_Sheet_1.docx]

Supplementary Material

**High-throughput phenotyping of sorghum plant height using an unmanned aerial vehicle and its application to genomic prediction modelling**

**Kakeru Watanabe, Wei Guo, Keigo Arai, Hideki Takanashi, Hiromi Kajiya-Kanegae, Masaaki Kobayashi, Kentaro Yano, Tsuyoshi Tokunaga, Toru Fujiwara, Nobuhiro Tsutsumi, Hiroyoshi Iwata^*^**

*** Correspondence:** Hiroyoshi Iwata: [aiwata@mail.ecc.u-tokyo.ac.jp](mailto:aiwata@mail.ecc.u-tokyo.ac.jp)


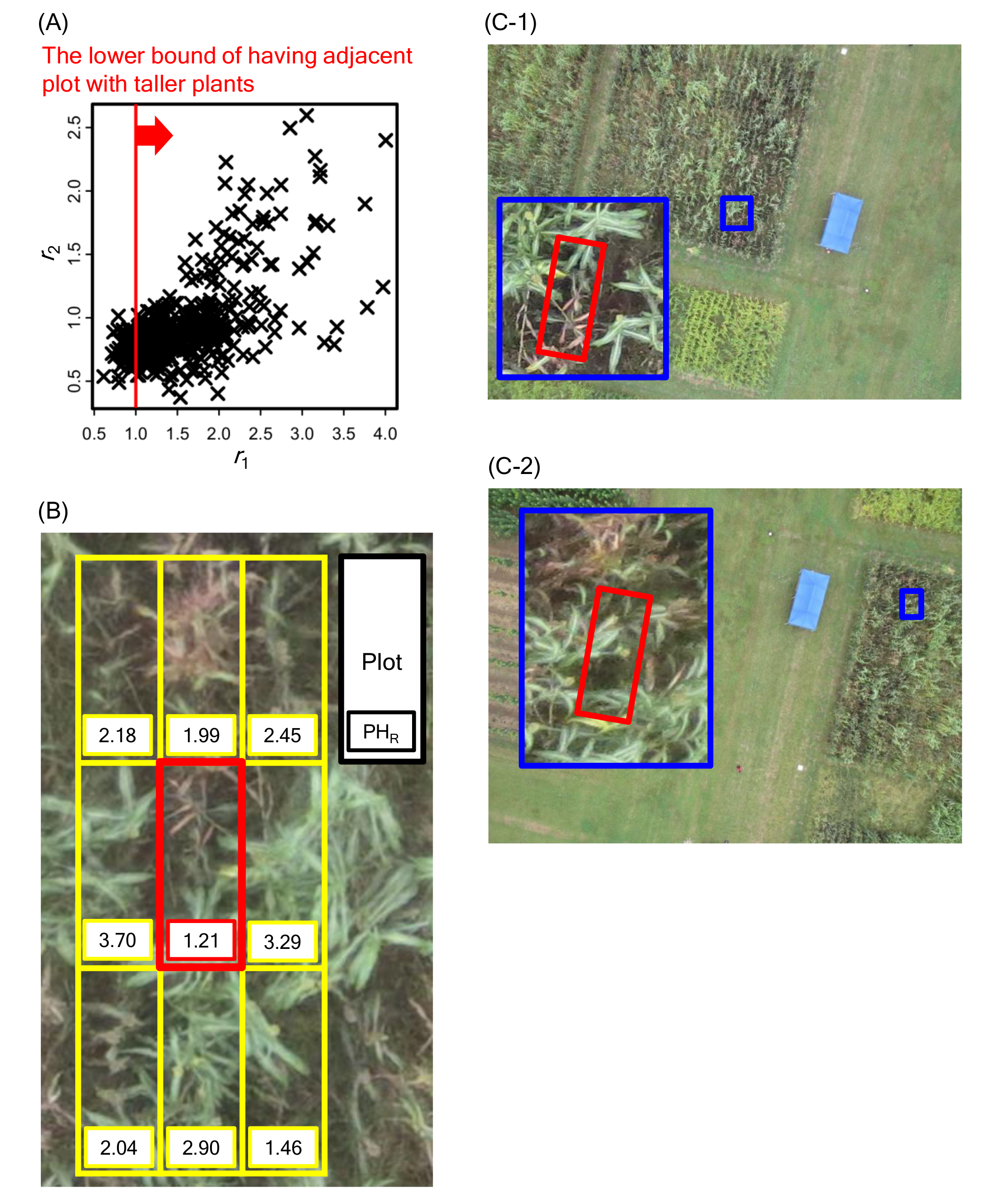


**Supplementary Figure 1 Effect of overlapping by taller plants from an adjacent plot.** (A) Scatter plot of *r*_1_ and *r*_2_ for the combination of NIR-GB and the 50^th^ percentile, where *r*_1_ = max{PH_R,_*_i_*_,_ *_k_* | *k* = 1, 2, ... , 8} / PH_R,_ *_i_*, and *r*_2_ = PH_UAV,_ *_i_* / PH_R,_ *_i_*; PH_R,_ *_i_* and PH_UAV,_ *_i_* are the PH_R_ and PH_UAV_ values of the *i*^th^ plot, respectively, and PH_R,_ *_i_*_,_ *_k_* (*k* = 1, 2, ... , 8) are the PH_R_ values of eight plots adjacent to the *i*^th^ plot. (B) A disturbed part of the ortho-mosaic around a plot with a strong overlapping effect (red rectangle; *r*_2_ = 2.59). (C-1) An image in which the target plot is near the center

of it. Individual plants can be seen. Some leaves belong to plants from adjacent plots. (C-2) An image in which the target plot is near the edge of it. Plants of the plot cannot be seen because they overlap with plants from an adjacent plot.

**Supplementary Table 1 Sorghum accessions used in this study.** We cultivated 172 accessions, investigated DNA polymorphism (SNP data) in 151 accessions and used these data to validate genomic prediction modelling.

| ID | | Cultivar name | Country of origin | SNP data |
| --- | --- | --- | --- | --- |
| **Sorghum world core collection of NIAS** | | | | |
|  | WSC001 | OOTOYO-MURA ZAIRAI | Japan | ○ |
|  | WSC002 | TAKAKIMI | Japan | ○ |
|  | WSC003 | IKEDACHO MATSUO ZAIRAI | Japan | ○ |
|  | WSC004 | KOUCHI OUKAWA ZAIRAI | Japan | ○ |
|  | WSC006 | TOKIBI | Japan | ○ |
|  | WSC007 | HIMEKI ZAIRAI | Japan | ○ |
|  | WSC008 | KIKUCHI ZAIRAI | Japan | ○ |
|  | WSC009 | AKAHO | Japan | ○ |
|  | WSC010 | KANAGAWAZAIRAI | Japan | ○ |
|  | WSC011 | 72-10-10-5 | Japan | ○ |
|  | WSC012 | HANGETSUTOSUI | Korea | ○ |
|  | WSC013 | KOUSHUU ZAIRAISHU | Korea | ○ |
|  | WSC014 | CHAL WAXY SORGHUM | Korea | ○ |
|  | WSC015 | KOUBOUSHI | Korea | ○ |
|  | WSC018 | CHOONCHAN LOCAL | Korea | ○ |
|  | WSC019 | 1972/8/13 | Taiwan | ○ |
|  | WSC020 | AI HUI | China | ○ |
|  | WSC021 | NUO GAO LIANG | China | ○ |
|  | WSC023 | LIAOZA 1 | China | ○ |
|  | WSC024 | BIG WHITE HULL | China | ○ |
|  | WSC026 | BATTANBAN | Cambodia | ○ |
|  | WSC027 | AS 5781 HUAN SA PHAUNG AH LPYSU | Myanmar | ○ |
|  | WSC029 | KALJANPUR | India | ○ |
|  | WSC031 | GOOSENECK | India | ○ |
|  | WSC032 | MARIANGARI JORA MUDDAHIHAL | India | ○ |
|  | WSC033 | DHOOTI ANEHULA | India | ○ |
|  | WSC034 | RABI YANGAR JORA MITHUGADUR | India | - |
|  | WSC035 | AS 4136 MASAKA LUWEMEA | India | ○ |
|  | WSC037 | COL/PAK/1991/IBPGR/2724(2) | Pakistan | ○ |
|  | WSC038 | COL/PAK/1989/IBPGR/2420(1) | Pakistan | ○ |
|  | WSC040 | COL/PAK/1989/IBPGR/2439(1) | Pakistan | ○ |
|  | WSC041 | COL/PAK/1989/IBPGR/2444(1) | Pakistan | ○ |
|  | WSC042 | COL/PAK/1989/IBPGR/2550(1) | Pakistan | ○ |
|  | WSC043 | COL/PAK/1989/IBPGR/2553(4) | Pakistan | ○ |
|  | WSC044 | COL/PAK/1989/IBPGR/2411(1) | Pakistan | ○ |
|  | WSC045 | COL/PAK/1989/IBPGR/2416(2) | Pakistan | ○ |
|  | WSC046 | COL/PAK/1989/IBPGR/2592(7) | Pakistan | - |
|  | WSC047 | 87-9-21-3-1 | Pakistan | ○ |
|  | WSC048 | 87-9-21-3-2 | Pakistan | - |
|  | WSC050 | PI 220636 Q 2/3/56 | Afghanistan | ○ |
|  | WSC051 | ALLAKH | Bangladesh | ○ |

**Supplementary Table 1** (continued)

| ID | | Cultivar name | Country of origin | SNP data |
| --- | --- | --- | --- | --- |
|  | WSC053 | JUNELO | Nepal | ○ |
|  | WSC054 | PI 229486 VULGARE | Iran | ○ |
|  | WSC056 | E 9 | Chad | ○ |
|  | WSC057 | PI 282834 | Chad | - |
|  | WSC058 | E 17 | Chad | ○ |
|  | WSC059 | MAKHOTLONG I | Lesotho | ○ |
|  | WSC060 | TENANT WHITE | Lesotho | ○ |
|  | WSC061 | NYAKASOBA BEST | Lesotho | ○ |
|  | WSC064 | KOURNIANIA | Morocco | ○ |
|  | WSC065 | PHATSAI | Morocco | ○ |
|  | WSC066 | SCHROCK | Morocco | ○ |
|  | WSC067 | ESHOME | South Africa | ○ |
|  | WSC071 | RED KAFIR | South Africa | ○ |
|  | WSC073 | EAR FROM PIETESBURG DL/60/107 | South Africa | ○ |
|  | WSC074 | MILO PET. 139/51 EX TANGANYIKA | Central Africa | ○ |
|  | WSC076 | 143 DINDERAWI 1 | Sudan | ○ |
|  | WSC077 | REDBINE 655 | Sudan | ○ |
|  | WSC080 | DINDERAWI 1 | Sudan | - |
|  | WSC081 | 240 WAD UMM BENEIN | Sudan | ○ |
|  | WSC082 | MUGBASH WHITE | Sudan | ○ |
|  | WSC084 | E 1091 | Sudan | ○ |
|  | WSC086 | ZA113 DAWA PAS PARA | Nigeria | ○ |
|  | WSC087 | AS 4547 JARDIRA | Nigeria | ○ |
|  | WSC089 | KA 24 | Nigeria | ○ |
|  | WSC090 | MN 401 | Algeria | ○ |
|  | WSC091 | S. VULGARE 72-726-7 | Uganda | ○ |
|  | WSC092 | S. VULGARE 72-728-1 | Uganda | ○ |
|  | WSC093 | E 276 FRAMIDA | Uganda | ○ |
|  | WSC094 | UGANDA L1 | Uganda | ○ |
|  | WSC097 | SC112 | Ethiopia | ○ |
|  | WSC100 | AKLMOI WHITE | Kenya | ○ |
|  | WSC102 | PI 152748 C | Kenya | ○ |
|  | WSC103 | WAD YABOO 132/53 | Zimbabwe | ○ |
|  | WSC104 | CAPE COLO 28/53 | Zimbabwe | ○ |
|  | WSC105 | TSETA LOCAL NATURE TYPE 27/51 | Zimbabwe | ○ |
|  | WSC106 | AS 4637 NHORONGO NENPI | Tanzania | ○ |
|  | WSC107 | E 37 | Tanzania | ○ |
|  |  |  |  |  |
| **Sorghum mini core collection of ICRISAT** | | | | |
|  | ICR003 | IS 603 | United States of America | - |
|  | ICR004 | IS 608 | United States of America | ○ |
|  | ICR008 | IS 1212 | China | ○ |
|  | ICR012 | IS 2382 | South Africa | ○ |

**Supplementary Table 1** (continued)

| ID | | Cultivar name | Country of origin | SNP data |
| --- | --- | --- | --- | --- |
|  | ICR014 | IS 2397 | South Africa | ○ |
|  | ICR018 | IS 2872 | Egypt | - |
|  | ICR020 | IS 3121 | Kenya | ○ |
|  | ICR026 | IS 4372 | India | ○ |
|  | ICR033 | IS 5094 | India | - |
|  | ICR034 | IS 5295 | India | - |
|  | ICR035 | IS 5301 | India | - |
|  | ICR042 | IS 7131 | Uganda | ○ |
|  | ICR050 | IS 8348 | Pakistan | ○ |
|  | ICR052 | IS 8777 | South Africa | ○ |
|  | ICR053 | IS 8916 | Uganda | - |
|  | ICR054 | IS 9108 | Kenya | ○ |
|  | ICR055 | IS 9113 | Kenya | - |
|  | ICR061 | IS 10969 | United States of America | ○ |
|  | ICR066 | IS 12302 | Zimbabwe | ○ |
|  | ICR068 | IS 12697 | Australia | ○ |
|  | ICR078 | IS 13782 | South Africa | ○ |
|  | ICR079 | IS 13893 | South Africa | ○ |
|  | ICR081 | IS 13971 | South Africa | ○ |
|  | ICR082 | IS 14010 | South Africa | - |
|  | ICR083 | IS 14090 | Argentina | ○ |
|  | ICR084 | IS 14290 | Botswana | ○ |
|  | ICR093 | IS 16151 | Cameroon | ○ |
|  | ICR096 | IS 17941 | India | ○ |
|  | ICR099 | IS 19153 | Sudan | ○ |
|  | ICR101 | IS 19389 | Bangladesh | ○ |
|  | ICR102 | IS 19445 | Botswana | ○ |
|  | ICR103 | IS 19450 | Botswana | ○ |
|  | ICR104 | IS 19676 | Zimbabwe | - |
|  | ICR105 | IS 19859 | India | ○ |
|  | ICR111 | IS 20679 | United States of America | - |
|  | ICR112 | IS 20697 | United States of America | ○ |
|  | ICR113 | IS 20713 | United States of America | ○ |
|  | ICR114 | IS 20727 | United States of America | ○ |
|  | ICR116 | IS 20743 | United States of America | ○ |
|  | ICR117 | IS 20816 | United States of America | ○ |
|  | ICR120 | IS 21512 | Malawi | ○ |
|  | ICR121 | IS 21645 | Malawi | ○ |
|  | ICR123 | IS 22239 | Botswana | ○ |
|  | ICR124 | IS 22294 | Botswana | ○ |
|  | ICR126 | IS 22616 | Myanmar | ○ |
|  | ICR127 | IS 22720 | Somalia | ○ |
|  | ICR128 | IS 22799 | Somalia | ○ |
|  | ICR131 | IS 23514 | Ethiopia | ○ |

**Supplementary Table 1** (continued)

| ID | | Cultivar name | Country of origin | SNP data |
| --- | --- | --- | --- | --- |
|  | ICR132 | IS 23521 | Ethiopia | ○ |
|  | ICR133 | IS 23579 | Ethiopia | ○ |
|  | ICR134 | IS 23586 | Ethiopia | ○ |
|  | ICR135 | IS 23590 | Ethiopia | ○ |
|  | ICR143 | IS 24348 | India | ○ |
|  | ICR145 | IS 24462 | South Africa | - |
|  | ICR146 | IS 24463 | South Africa | ○ |
|  | ICR147 | IS 24492 | South Africa | ○ |
|  | ICR164 | IS 26694 | South Africa | ○ |
|  | ICR165 | IS 26701 | South Africa | ○ |
|  | ICR166 | IS 26737 | South Africa | ○ |
|  | ICR167 | IS 26749 | South Africa | ○ |
|  | ICR175 | IS 28313 | Yemen | ○ |
|  | ICR176 | IS 28389 | Yemen | ○ |
|  | ICR178 | IS 28451 | Yemen | ○ |
|  | ICR184 | IS 29187 | Swaziland | ○ |
|  | ICR185 | IS 29233 | Swaziland | ○ |
|  | ICR187 | IS 29241 | Swaziland | ○ |
|  | ICR188 | IS 29269 | Swaziland | ○ |
|  | ICR189 | IS 29304 | Swaziland | ○ |
|  | ICR190 | IS 29314 | Swaziland | ○ |
|  | ICR191 | IS 29326 | Swaziland | ○ |
|  | ICR192 | IS 29335 | Swaziland | ○ |
|  | ICR194 | IS 29392 | Lesotho | ○ |
|  | ICR195 | IS 29441 | Lesotho | ○ |
|  | ICR196 | IS 29468 | Lesotho | - |
|  | ICR197 | IS 29519 | Lesotho | ○ |
|  | ICR199 | IS 29568 | Lesotho | ○ |
|  | ICR200 | IS 29582 | Lesotho | ○ |
|  | ICR201 | IS 29606 | South Africa | ○ |
|  | ICR202 | IS 29627 | South Africa | ○ |
|  | ICR204 | IS 29689 | Zimbabwe | ○ |
|  | ICR205 | IS 29714 | Zimbabwe | ○ |
|  | ICR206 | IS 29733 | Zimbabwe | ○ |
|  | ICR210 | IS 30079 | Zimbabwe | ○ |
|  | ICR211 | IS 30092 | Zimbabwe | ○ |
|  | ICR212 | IS 30231 | Zimbabwe | ○ |
|  | ICR220 | IS 30466 | China | ○ |
|  | ICR223 | IS 30533 | Korea | ○ |
|  | ICR226 | IS 30572 | Cameroon | - |
|  | ICR233 | IS 31681 | Algeria | ○ |
|  | ICR238 | IS 31706 | Yemen | - |
|  | ICR239 | IS 32787 | Somalia | ○ |

**Supplementary Table 1** (continued)

| ID | | Cultivar name | Country of origin | SNP data |
| --- | --- | --- | --- | --- |
| **Original cultivars developed by EARTHNOTE Co., Ltd.** | | | | |
|  | UTSb4002 | - | - | ○ |
|  | UTSb4040 | - | - | - |
|  | UTSb4105 | - | - | - |
